# A first online intervention to increase patients’ perceived ability to act in situations of abuse in health care: reports of a Swedish pre-post study

**DOI:** 10.1186/s12910-015-0027-7

**Published:** 2015-05-24

**Authors:** A. Jelmer Brüggemann, Katarina Swahnberg, Barbro Wijma

**Affiliations:** Department of Clinical and Experimental Medicine, Gender and Medicine, Linköping University, 581 83 Linköping, Sweden; Department of Health and Caring Sciences, Faculty of Health and Life Sciences, Linnaeus University, 392 34 Kalmar, Sweden; Current Address: Department of Thematic Studies – Technology and Social Change, Linköping University, 581 83 Linköping, Sweden

**Keywords:** Abuse in health care, Patient intervention, Online intervention, Patient empowerment, Comics

## Abstract

**Background:**

Efforts to counteract abuse in health care, defined as patient-experienced abuse, have mainly focused on interventions among caregivers. This study is the first to test an online intervention focusing on how patients can counteract such abuse. The intervention aimed at increasing patients’ intention and perceived ability to act in future situations where they risk experiencing abuse.

**Methods:**

Participants were recruited through a nephrology clinic in Sweden. The intervention consisted of an online program that aimed to stimulate patients to think of possible actions in situations in which they risk experiencing abuse. The program comprised stories and exercises in text and comic form. The participants filled out a questionnaire immediately before and after going through the program, as well as during follow-up four to eight weeks later.

**Results:**

Forty-eight patients (39 %) participated in the study and spent, on average, 41 min responding to questions and going through the program. Both men and women, of various ages and educational backgrounds, participated. An increase in participants' self-reported ability to identify opportunities to act in a given situation was seen immediately afterwards, as well as during follow up.

**Conclusion:**

The current study suggests that it is feasible and most likely useful to a variety of patients to work with the provided material that has the aim of counteracting abuse in health care. It would be of interest to further develop ways of using comics and to test similar interventions in other health care settings.

## Background

Interventions against abuse as experienced by patients in different health care settings have mostly involved actors who are responsible for the quality of care: health care professionals, hospital management, and policymakers. Examples of such interventions include the introduction or revision of behavioural or ethical guidelines [1–3], workshops to change nurses’ attitudes [2], or workshops to protect staff’s moral resources [4]. Patients have not been invisible in these efforts, but have mainly been seen as (potential) victims or subjects of abuse, and the focus has been on their ability to cope and their need for support [5], and not on the patient as a potent actor. If, however, the abuse is seen as an interactional problem embedded in a complex structural and cultural setting [2, 6, 7], then it is of utmost importance to include all actors, even patients [8]. In this article, we report on a first intervention against abuse in health care, defined as events that patients experience as abuse, and where patients are involved as actors.

There are several ways in which patients can be harmed as a result of the health care that they receive. One large category of incidents that have received increasing attention is medical errors [9]. These errors, ranging from near-misses to fatal accidents, are always defined by the medical community; patients never decide what is a medical error and what is not. This stands in contrast to another group of harmful incidents that consist of abusive and unethical treatment. Such abuse is subjectively defined from the patients’ perspective and can occur despite the lack of any medical mistakes [6]. If patients experience abusive treatment in relation to a medical mistake, this can lead to worse outcomes compared to receiving respectful treatment. Compared to medical mistakes, incidents of abuse in health care are much less studied, despite their potential for serious patient suffering. Perhaps this can be explained by “an underlying belief held by many within society that ‘health care professionals do not abuse’” ([10], p. 1152). However, using the NorVold Abuse Questionnaire [11], it was found that 20 % of female patients and 8 % of male patients in Sweden reported experiences of abuse in health care, and a majority were still suffering from the experience [12, 13]. In Swedish qualitative studies, female patients described the experience of abuse as “being nullified” [14] and male patients felt “mentally pinioned” [15], both indicating a serious threat to patients’ human value. Patients’ experiences cover a wide range of abuses and ethical transgressions, including episodes of neglect, exclusion, ignorance, humiliation, and physical and sexual maleficence [16]. Studies indicate that abuse is a phenomenon present in health care settings around the world [2, 17, 18], although what patients experience as abusive may differ according to the cultural, structural, and historical context.

Incidents of abuse can be among those that are most damaging to quality of care and considering the prevalence of abuse and the impact it can have on patients’ lives, we felt an urgent need to develop ways to tackle this ethical problem.

### The intervention model

The current intervention had its origin in research about how patients respond to abusive and untoward health care encounters. It seems to be the norm rather than the exception that patients remain silent and take little action towards health professionals after untoward or abusive experiences in health care [16, 19, 20]. An important consequence of this “norm of silence” is that structural settings are being reproduced without alteration, nurturing possible future incidents, especially if the events go by unnoticed by staff. This silence has been explained by a variety of factors, most of them pointing in the direction of patients’ feelings of powerlessness [15, 19]. We assumed that a decrease in patients’ feelings of powerlessness can be an important mechanism for intervention, for two reasons. First, if patients are empowered to act or use their voice, that can protect them from further suffering. Second, patients’ increased level of action can be an important source of feedback for staff, as it can not only visualise the (unforeseen) consequences of staff’s actions, but also offer them an opportunity to repair the damage and alter their future behaviour [21]. Any such intervention should focus on opportunities for change in the patients’ interest, rather than on a transfer of responsibility for the quality of care from professionals to patients.

The intervention model that was chosen was inspired by a staff intervention against abuse in health care based on Forum Play [4]. Forum Play has its roots in Augusto Boal’s interactive Theatre of the Oppressed, which he developed as a tool for liberation in situations of oppression [22, 23]. One important element in Boal’s pedagogy is that the oppressed can learn and feel empowered by seeing and testing a variety of possible behavioural paths in problematic situations, often provided by the oppressed themselves. Instead of testing this theatre model directly in patients, we used some of its core ideas to develop an online intervention program that would be easier to implement and less resource intensive.

**Fig. 1:**
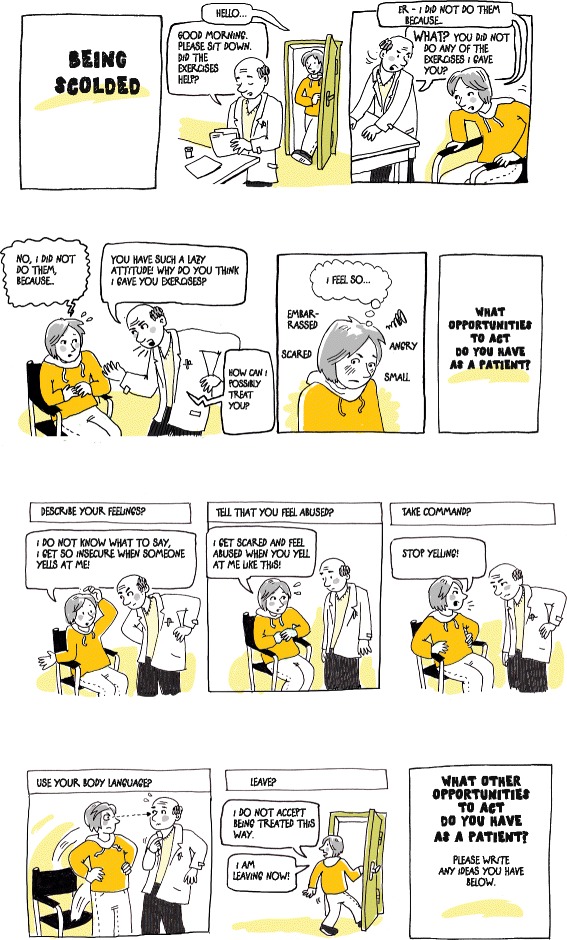
One of the three comics used in the online program. Copyright © 2013–14 Ka Schmitz. Used with permission

The content of the program (Table 1) was based on clinical experiences and our knowledge of abusive situations from previous studies on patients and staff [14–16, 20, 24–28]. Explorative interviews with two members of staff and two patients from the intervention clinic were conducted in order to adapt the program to the current patient group. The program was developed in collaboration with a drama instructor specialising in Forum Play and an activist artist who had experience of using comics to activate bystanders in situations of potential violence (Fig. 1).

**Table 1:** Content of the online program in order of appearance on the website

Part of the program	Description	Objective
Pre-intervention questions	See Methods section	
Research about abuse in health care	A short summary of studies about abuse in health care.	To provide a starting point and to legitimise the participants’ own experiences of abuse.
Participants’ experiences	Participants get the opportunity to write down their own experiences of abuse in health care.	To get participants to start thinking about abusive situations, and perhaps recall their own experiences. Also, to gather stories as research material for future studies.
Text scenarios	Two short scenarios constructed from real situations. Both cases end with a patient feeling terribly offended and embarrassed. Participants are then asked to imagine ending up in a similar situation and to think of things they, as patients, could do to protect themselves, find a way out of the situation, or turn the situation in a different direction. Participants are then asked to write down what possibilities they have identified.	To familiarise participants with the idea that there could be possibilities for them to act, as well as to let them think of different possibilities, and to increase their and our understanding of what can be abusive in health care settings.
Comics	Three scenarios in comic form constructed from real situations are shown. All three scenarios picture a story in about five frames, the last frame showing a patient feeling devastated, followed by the question, “What opportunities to act do you have as a patient?” On the next page, a few suggestions are shown in additional frames. After these suggestions, participants are asked to write down what other possibilities they have identified (see Fig. 1).	In addition to objectives similar to those of the text scenarios, the element of suggestions aimed to simulate possibilities provided by other participants, as done in Forum Play, and to stimulate participants to think in different directions. The visual aspect was assumed to bring stories and characters to life without excessive amounts of text and to provide a reality in which alternative consequences can be explored [39, 40].
Stories from earlier patients	Two short stories written by two female patients who had experienced abuse in different health care settings. These stories include details about the incidents and the patients’ own reflections upon what they did or could have done themselves in the situations they described.	To legitimise further feelings related to abuse that the participants may carry with them, and to offer some insights in how other patients reflect upon their actions in situations of abuse.
Staff stories	One short story written by a female health professional about an episode in which she was confronted by a patient who had felt abused and which led to changes in work routines.	To give an example of how a patient’s actions, in response to treatment that she experienced as abusive, had affected staff’s future routines (which may not always be visible to patients).
Post-intervention questions	See Methods section	
Thank you	A link to a detailed summary of research about abuse in health care and a comprehensive overview of possibilities for patients who have experienced abuse in health care and wish to express grievances or file a complaint.	To give information for patients who are interested in the topic. Also, to give patients tools to deal with events that they thought of during the interventions.

### Hypotheses

Besides exploring the feasibility of the intervention in terms of participation (degree, distribution among different patient groups), the main hypotheses guiding the intervention were that it would strengthen patients’ perceived ability and intention to act in situations of abuse in health care, and generally increase their ability to identify opportunities to act.

## Methods

### Procedure and participants

Between September 2013 and January 2014, patients visiting a nephrology clinic at a hospital in the south of Sweden were informed of the study via posters and information letters. Staff also informed patients coming for dialysis or for an outpatient visit about the ongoing study and referred them to the information letter. On this letter, patients had the opportunity to leave their email address if they were interested in receiving an invitation to participate. Information letters with email addresses were collected in a box next to the reception desk and staff regularly sent the letters to the first author. All patients who had left their email address received a formal invitation to the study by email, including a link to a consent page and a unique ID number, which was necessary to access the program. On the consent page, patients obtained all necessary instructions and detailed information about the study and were then offered a choice between “consent and participate” and “not participate”. A reminder was sent out after two weeks. Four to eight weeks after participation, a link to a follow-up questionnaire was sent out by email to participants who had completed the program. In addition, a reminder was sent out after two to four weeks. The study was approved by the regional ethical review board in Linköping, Sweden (reg. no. 2013/242-31).

### Measurements

The following measurements were used to evaluate the intervention model.

- Pre-intervention (first page on website). The questionnaire started with questions covering sex, age, education, native language, and the MacArthur Scale of Subjective Social Status [29, 30]. After that, the participants were asked about their lifetime experiences of abuse in health care according to three validated questions from the NorVold Abuse Questionnaire (Table 2) [11, 31]. The last set of questions consisted of the Intention to act in Situations of Abuse in Health Care Questionnaire (ISAHCQ), aiming at capturing participants’ intention and perceived ability to act in situations of abuse in health care (Table 3). Different self-efficacy scales were considered but were deemed too general and, as no other suitable instrument was available, ISAHCQ was constructed within the project. Therefore, psychometrically, this study should be seen as a pilot, comprising a first attempt to develop an appropriate instrument. ISAHCQ is based on the theory of planned behaviour, which has been widely used in the study of health behaviour and behavioural change, assuming that attitudes, subjective norms, and perceived behavioural control (Ajzen’s term for self-efficacy) can predict an individual’s intention to perform a central behaviour [32, 33]. ISAHCQ only included specific variables that we assumed could be affected by the intervention, as suggested in a manual [33]. ISAHCQ starts with a case description and also includes an example of what is meant by “acting” (Table 3). ISAHCQ aims to capture participants’ intentions (item 1), attitudes (items 2, 3, and 4), and perceived behavioural control (items 5 and 6). After reversing the score for items 2, 3, and 4 (0 = 10, 10 = 0), all items are summed in order to obtain a total ISAHCQ score varying from 0 to 60. A high ISAHCQ score was assumed to represent a greater likelihood that the participant would act in the given scenario.
- Post-intervention (last page on website). Here, the participants once more filled out ISAHCQ and rated their subjective judgement of the effect of the intervention: “This website aimed to strengthen patients’ ability to act in case they experience abuse in health care situations. To what degree do you think this aim was achieved for you?” (0, not at all – 10, completely). The participants had to estimate changes in their perceived ability because they had no experiences on which to base their answer. Participants also answered questions about the general quality of the material and the layout of the website.
- Follow-up (four to eight weeks later). All participants who had completed the program once again filled out ISAHCQ and answered questions covering their subjective judgement of the effect of the intervention. The participants also reported the number of times they had spoken about abuse in health care with staff or people in their surroundings, and the number of times they had heard of any stories about abuse in health care, during the last month and the month before the intervention. Lastly, participants were asked whether they would recommend the website to other patients, if it were to become available to them.

**Table 2:** Three validated questions about abuse in health care in the NorVold Abuse Questionnaire. To estimate prevalence rates, abuse in health care was operationalised as at least one ‘Yes’ to one of the three questions

Mild abuse	Have you ever felt offended or grossly degraded while visiting health care services, felt that someone exercised blackmail against you or did not show respect for your opinion – in such a way that you were later disturbed by or suffered from the experience?
Moderate abuse	Have you ever experienced that a “normal” event, while visiting health care services, suddenly became a really terrible and insulting experience, without you fully knowing how this could happen?
Severe abuse	Have you ever experienced anybody in health service purposely – as you understood – hurting you physically or mentally, grossly violating you or using your body and your subordinated position to your disadvantage for his/her own purpose?
	Answer alternatives (same for all questions): 1 = No, 2 = Yes, as a child (<18 years), 3 = Yes, as an adult (≥18 years), 4 = Yes, as a child and as an adult.

**Table 3:** The Intention to act in Situations of Abuse in Health Care Questionnaire (ISAHCQ), based on the theory of planned behaviour [33]

**Below, a situation is presented where you are the patient.**			
During an eye test, the optician finds a splinter in your eye and advises you to seek help at a medical centre. According to the optician, a GP should be able to see the splinter with a specific kind of microscope and remove it. Your GP takes a look in your eye, but does not see anything and is about to send you home. You then say what the optician had told you, namely, that the splinter can be seen with a microscope of a certain kind. The GP then starts getting mad at you for telling him how to perform the examination – and yells at you. You think the situation is really uncomfortable and start feeling as small as an ant.			
The questions below refer to acting in this situation. By the term “acting”, we mean actively doing something in this situation, for example, making it clear to the GP how you perceive his behaviour. Below, you find a number of statements and we ask you to choose the number that best corresponds to what you think or experience.			
1. I find it likely that I would act in this situation	Disagree	0 1 2 3 4 5 6 7 8 9 10	Agree
2. It is meaningless to act in this situation	Disagree	0 1 2 3 4 5 6 7 8 9 10	Agree
3. It is uncomfortable to act in this situation	Disagree	0 1 2 3 4 5 6 7 8 9 10	Agree
4. As a patient, I am powerless in this situation	Disagree	0 1 2 3 4 5 6 7 8 9 10	Agree
5. I can easily identify opportunities for me to act in this situation	Disagree	0 1 2 3 4 5 6 7 8 9 10	Agree
6. Generally, I am confident that I would be able to act in this situation	Disagree	0 1 2 3 4 5 6 7 8 9 10	Agree

### Analysis

In order to test the main hypotheses, for total ISAHCQ scores as well as for single items, differences between pre-, post-, and follow-up measurements were analysed by a non-parametric test for paired ordinal data (Wilcoxon signed-rank test). Statistical analyses were performed with SPSS version 22.0. Test results with a *p*-value below 0.05 were deemed statistically significant.

## Results

### Participation

**Fig. 2:**
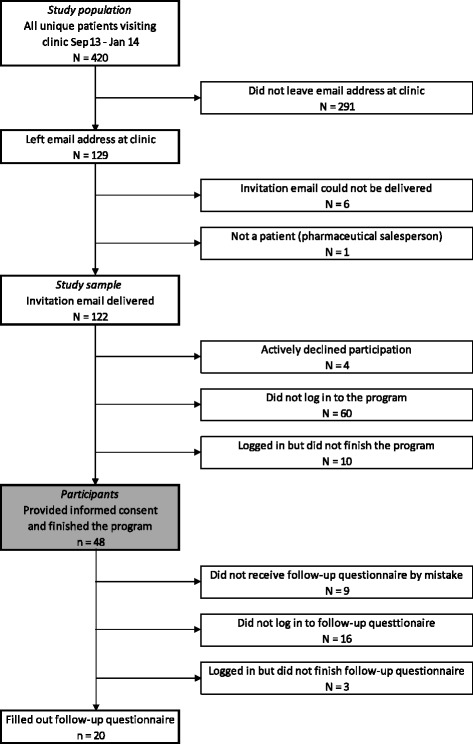
Flow chart representing the inclusion of patients. The area shaded grey represents the participants

Of all the patients who received an invitation to participate, 48 % (58/122) started the program and 39 % (48/122) finished it (hereafter referred to as “participants”, Fig. 2). The group of participants showed large variation in terms of their background characteristics (Table 4).

**Table 4:** Background characteristics of the participants

		Participants (n = 48)^a^	
	Mean/median and range	n	%
Age			
	mean 56.0 year ± SD 14.2 years, range 23–81 year		
Sex			
Female		22	49.0
Male		23	51.0
Education (years)			
Primary (<10)		9	19.1
Secondary (10–12)		25	53.2
Higher (>12)		13	27.7
Subjective social status^b^			
	Median 6, range 1–10		
Native language			
Swedish		42	89.4
Other		5	10.6
Any lifetime abuse in health care^c^			
No		30	63.8
Yes		17	36.2

^a^Not all values of ‘n’ add up to 48 due to item non-response

^b^According to the MacArthur Scale of Subjective Social Status (0–10, 10 being the highest)

^c^An answer of "yes" to any one of three questions from the the Norvold Abuse Questionnaire

As nothing was known about the patients who did not participate, no conclusions about systematic non-response could be drawn. No associations were found between “days to respond” (the number of days between invitation and participation) and key variables such as total ISAHCQ score and having experienced abuse in health care. This could indicate that participants did not differ from the study population with regard to these two variables [34]. The rate of non-response to items in the questionnaire ranged from 0 to 9 %, which was considered low.

Due to an administrative error, nine participants did not receive the follow-up questionnaire. These nine participants did not differ from the other participants in terms of their background characteristics or outcome variables, but had taken considerably more days to proceed from invitation to participation (mean: 19 days compared with 5 days, ANOVA, p < 0.01). The response rate to the follow-up measurement was considered reasonable as 20 out of 39 (51 %) participants who received an invitation filled out the entire questionnaire. Non-response was not related to background characteristics, but those who responded had lower pre-intervention ISAHCQ scores (Mann–Whitney *U* test, p = 0.03) and greater differences between post- and pre-intervention ISAHCQ scores (Mann–Whitney *U* test, p = 0.04). This could indicate that attrition at this stage was related to low perceived relevance and/or effect of the program.

### Intention to act

Participants reported that the online program had achieved its aim of increasing their perceived ability to act to a high degree (median rating: 7). However, this was not equally apparent in analyses of the participants’ ISAHCQ scores at a group level. Pre-intervention ISAHCQ scores ranged from 15 to 60 (median: 46), post-intervention ISAHCQ scores ranged from 22 to 60 (median: 47), and follow-up ISAHCQ scores ranged from 17 to 60 (median: 48). However, participants with pre-intervention ISAHCQ scores below the 50th percentile showed an increase in ISACHQ scores at post-intervention (Wilcoxon signed-rank test, r = 0.51, p = 0.03). In addition, within the group of participants who responded to the follow-up questionnaire, there was rather strong evidence for an increase in ISAHCQ scores upon comparing follow-up with pre-intervention scores (p = 0.05, Table 5). An analysis of the effects only within this group also indicated evidence for an increase in ISAHCQ scores from pre- to post-intervention (p = 0.07, data not shown).

**Table 5:** ISAHCQ scores post-compared with pre-intervention, and follow-up compared with pre-intervention

Participants (n = 48)^a^	Post - pre (n = 48)		Follow-up - pre (n = 20)	
ISAHCQ items	*Z* ^b^	*p*-value	*Z* ^b^	*p*-value
1. I find it likely that I would act in this situation	−1.34	0.18	−0.49	0.62
2. It is meaningless to act in this situation	−1.47	0.14	−0.10	0.92
3. It is uncomfortable to act in this situation	−0.20	0.84	−0.77	0.44
4. As a patient, I am powerless in this situation	−1.30	0.19	−0.95	0.34
5. I can easily identify opportunities for me to act in this situation	−2.46	0.01^c^	−2.31	0.02^d^
6. Generally, I am confident that I would be able to act in this situation	−1.00	0.32	−1.08	0.28
Total ISAHCQ score (items 1–6)	−1.37	0.17	−1.96	0.05

^a^Variations in ‘n’ exist due to non-response

^b^Wilcoxon signed-rank test

^c^r = 0.37

^d^r = 0.54

Differences were found in the most important ISAHCQ item for this study, namely, participants’ ability to identify opportunities to act, which had increased immediately after the intervention, and remained increased during follow-up (item 5, Table 5).

Experiences of abuse in health care were not associated with any ISAHCQ scores or any changes in these scores, but were related to pre-intervention scores on item 4: Patients with previous experiences of abuse in health care agreed to a higher degree that they were powerless in the described situation (Mann–Whitney U, p = 0.05). ISAHCQ scores were in no way associated with the participants’ sociodemographic background.

During the follow up, no differences were seen in the participants’ reporting of the number of times that they had spoken to (i) staff or (ii) others in their surroundings about abuse in health care, nor in (iii) the number of stories about abuse in health care that they had heard (last month compared to the month before the intervention, paired sample *t*-test, (i) p = 0.22, (ii) p = 0.55, and (iii) p = 0.10).

### Validity and reliability

Cronbach’s alpha values for ISAHCQ were found to be 0.75 and 0.77 at pre- and post-intervention, respectively, demonstrating good internal consistency. The rate of item non-response was considered low.

Total ISAHCQ scores as well as individual ISAHCQ items showed no correlation with the participants’ subjective judgement about the extent to which the website achieved its aim of strengthening their ability to act.

### Evaluation of the online program

In general, participants found working with the online program meaningful, instructive, and interesting, but some also found it a difficult and strenuous task. This is not to say that this is necessarily negative because reporting that the program was strenuous was highly correlated with an increase in total ISAHCQ score during the follow-up (Spearman’s ρ = 0.71, p < 0.01). The participants would generally recommend the program to other patients if it were to become available to them.

## Discussion

In this article, we report the findings of a first intervention study to increase patients’ perceived ability to counteract abuse in health care. It was encouraging to see that almost 40 % of the patients who received an invitation participated in the study and, on average, dedicated 41 min to work with and evaluate the program. In addition, a variety of patients, in terms of their gender, age, and educational background, showed an interest in the intervention material. The participants reported an increase in their self-estimated ability to identify opportunities to act in a given situation. Furthermore, the intervention seemed to be of most relevance to participants who initially reported a lower intention to act. However, this finding should be interpreted with caution as our main effect measure was not validated and needs further testing.

Our endeavour to explore the possibilities for patients to counteract abuse in health care can be seen as part of a larger patient empowerment movement. Patient empowerment builds on an ideology that is concerned with patients’ increased choices and responsibilities on the one hand, and patients’ skills on the other [35]. Patient empowerment can be understood on an inter-personal as well as intra-personal level [35]. By focusing on intentions and planned behaviour, we have mainly focused on the intra-personal level, on patients’ understanding of their own personal power [36]. However, what happens on an inter-personal level when patients decide to act or speak up in health care encounters in which they are at risk of being abused is pivotal for the continued empowerment process. In a study using qualitative interviews with patients about their experiences of abuse in health care, it seemed that staff had responded to the patients’ use of their competence and speaking up by using domination techniques [27]. This study signalizes that patients may have realistic fears that taking action may make things worse or mean that they do not receive necessary care. These experiences also emphasise the need for staff to learn how to respond to patients’ actions in these situations. Therefore, we also conducted a staff intervention against abuse in health care based on Forum Play parallel to the patient intervention at the same clinic, which will be reported elsewhere.

As we only built on some parts of Boal’s Forum Play pedagogy, others were necessarily removed, considering the nature of our intervention. In contrast to a Forum Play workshop, the participants were (i) alone instead of being in a group, and (ii) were encouraged to think and write, instead of act with their bodies. First, to compensate for being alone, we simulated options offered by others by presenting possible solutions in the comics. This approach does not compensate for the absence of cooperating and shared problem-solving activities, but can aid patients in developing more ideas. Second, even though there were potential losses through the lack of embodied experiences, cognitive exercises can still be a very powerful tool to promote behavioural changes. For comparison, so-called *imagined exposure* is a widely used technique in behavioural therapy, for example, if a real situation is not available [37]. This use of imagination most likely depends on how real the material is to the patient (as is the case in Boal’s theatre methods), and to what extent the patient can identify her- or himself with the characters, for example, those in the comics. For the current material, we talked to staff and patients from the test clinic in order to create situations that were recognisable and realistic to nephrology patients. In order to increase the likelihood that many patients would identify with the comics, we attempted to include a variety of characters in terms of age, gender, profession, and to some extent ethnicity. One way of doing this with a limited number of images was to develop a character with few gender specific attributes in order to enable identification with the character by as many people as possible (see Fig. 1).

The fact that both men and women, young and old, and people with different educational backgrounds participated in this study reflects well on the accessibility of the program. Compared with other male and female patient samples in Sweden, the prevalence of any lifetime experience of abuse in health care was much higher in the current sample (27 % among men and 50 % among women, compared with previous rates in Sweden of 8 % in male patients [13] and 20 % in female patients [12]). This difference could depend on the fact that the current patient group has visited health care facilities more often, as many nephrology patients suffer from chronic kidney failure or come for regular check-ups. More likely, however, is that this study, in contrast to earlier studies, was solely concerned with abuse in health care and therefore attracted a more specific patient group. Given the current study’s aims and considering the small sample size, this selection effect was not at all disadvantageous, as earlier experiences of abuse in health care are likely to affect patients’ thoughts about future health care encounters. The fact that the program was not equally relevant to all patients can possibly explain the attrition during the study period, which we find reasonable. Interpreting this attrition, it should also be borne in mind that the study sample consisted of many seriously ill patients, who may not have been able to participate, and older patients, who might not use computers on a daily basis. It should be noted, in general, that a certain level of informatics skills was needed in order to participate in the study, thereby limiting the generalizability of our study.

We do not know which part of the intervention affected the participants. We can assume the importance of the exercises themselves, but the legitimising effect of the intervention could also have been important. There is also a risk that the effects that we identified were influenced by a response bias, and more specifically by demand characteristics. The so-called “good-subject effect” could lead participants to respond in the way that they believe the researcher would want them to respond [38]. In the current study, it is not unlikely that the participants figured out which hypotheses were to be tested and which questions were indicators. However, when the participants filled out ISAHCQ for the first time, they did not know that they would answer the same questions again after the program. More importantly, after the program, there was no opportunity to view or alter responses to pre-intervention questions. The participants could still have remembered what they answered and used that as a baseline for post-intervention answers, but no such general tendency was seen in Table 5, as many items remained stable during the study period. It is possible, though, that a demand bias arose in the part in which participants were asked to evaluate the effect of the program as to whether it had achieved its aim of strengthening their self-estimated ability to act. This could explain why some reported that the program achieved its aim, despite no positive changes in ISAHCQ scores. Alternatively, it could also be that ISAHCQ does not capture the effect of the intervention in the same way in which the above general question does. The fact that ISAHCQ was not validated should therefore, once more, be borne in mind. Despite that, participants who reported greater differences between post- and pre-intervention ISAHCQ scores were more likely to respond to the follow-up measurement, which may indicate that ISAHCQ scores relate to perceived effects or relevance of the program. In addition, ISAHCQ showed good internal consistency, indicating that it in this study seemed to be a reliable measurement of the latent construct of participants’ intention to act. However, there is a need to further examine the instrument’s validity, for example in relation to established instruments in areas such as patient empowerment.

## Conclusion

This study is the first to report on an online patient intervention to counteract abuse in health care. It is suggested that it is possible and most likely useful to a varied group of patients to work with the intervention material that we provided. Such interventions may appeal most to patients who have experienced abuse or who find it unlikely that they would act in situations in which they risk being abused. However, further testing of intervention material and evaluation methods is needed. It would be of great interest to explore different ways of using comics and to test similar interventions in other patient groups, other health care settings, and in relation to different types of abuse, and we hope our results can inspire others to do so.
